# Hepatic Mitochondrial Alterations and Increased Oxidative Stress in Nutritional Diabetes-Prone *Psammomys obesus* Model

**DOI:** 10.1155/2012/430176

**Published:** 2012-05-17

**Authors:** Saida Bouderba, M. Nieves Sanz, Carlos Sánchez-Martín, M. Yehia El-Mir, Gloria R. Villanueva, Dominique Detaille, E. Ahmed Koceïr

**Affiliations:** ^1^USTHB, FSB, Equipe de Bioénergétique et Métabolisme Intermédiaire, Alger 16111, Algeria; ^2^Departamento de Fisiología y Farmacología, Faculad de Farmacia, Universidad de Salamanca, 37007 Salamanca, Spain; ^3^Laboratoire de Bioénergétique Fondamentale et Appliquée (LBFA), Université Joseph Fourier, Grenoble 38041, France; ^4^INSERM U1055, Grenoble cedex 9, Grenoble 38041, France

## Abstract

Mitochondrial dysfunction is considered to be a pivotal component of insulin resistance and associated metabolic diseases. *Psammomys obesus* is a relevant model of nutritional diabetes since these adult animals exhibit a state of insulin resistance when fed a standard laboratory chow, hypercaloric for them as compared to their natural food. In this context, alterations in bioenergetics were studied. Using liver mitochondria isolated from these rats fed such a diet for 18 weeks, oxygen consumption rates, activities of respiratory complexes, and content in cytochromes were examined. Levels of malondialdehyde (MDA) and gluthatione (GSH) were measured in tissue homogenates. Diabetic *Psammomys* showed a serious liver deterioration (hepatic mass accretion, lipids accumulation), accompanied by an enhanced oxidative stress (MDA increased, GSH depleted). On the other hand, both ADP-dependent and uncoupled respirations greatly diminished below control values, and the respiratory flux to cytochrome oxydase was mildly lowered. Furthermore, an inhibition of complexes I and III together with an activation of complex II were found. With emergence of oxidative stress, possibly related to a defect in oxidative phosphorylation, some molecular adjustments could contribute to alleviate, at least in part, the deleterious outcomes of insulin resistance in this gerbil species.

## 1. Introduction

The pathophysiology of type 2 diabetes mellitus (T2DM) is varied and very complex but the association of T2DM with obesity and inactivity indicates a potentially pathogenic link between fuel homeostasis, emergence of insulin resistance, and disease progression. Given the central role for mitochondria in energy production, dysregulated mitochondrial function at the cellular level can impact whole-body metabolism. Three major players are believed to be involved in such a disordered context: hepatocytes, insulin-dependent tissues (skeletal muscle, fat), and *β*-cells. Evidence pointing to defects in mitochondrial oxidative capacity for all these cell types demonstrates that each of them contributes to glucose imbalance [1]. Nevertheless, the nature, origin, and extent of this dysfunctioning remain controversial. Reduced expression of oxidative phosphorylation genes was observed in muscle and adipose tissue of humans with T2DM [2], while other studies reported on increases in respiration intensity of liver mitochondria from Goto-Kakizaki (GK) rats, a rodent model of diabetes [3], or diabetic patients [4]. In any case, it is important to realize that mitochondrial abnormalities could add to hyperglycemia once the insulin resistance is in place and lead to worsening of the diabetic state.

Due to its strategic position, between the intestinal bed and the systemic circulation, the liver was regarded as buffer organ for the regulation of metabolic fluxes [5, 6]. As glucose and fatty acid metabolisms are largely dependent on mitochondria to generate energy in cells, any impairment in nutrients oxidation, together with a reduced mitochondrial content, could thereby establish a vicious cycle of metabolic alterations involved in the pathogenesis of T2DM, leading to an increased generation of free radicals. Oxidative stress is recognized to contribute to many pathological processes, and a number of works point to the role of hyperglycaemia in promoting overproduction of mitochondria-derived reactive oxygen species (ROS) [7]. On the other hand, oxidative stress might also result from diminution of the antioxidative capacity in plasma and within the cells of diabetic subjects. Indeed, and in spite of more uncertain data, various reports shown that liver concentrations of reduced glutathione (GSH) decreased in different rodents models of diabetes [8, 9].

Several rodents models have been used to investigate the pathogenesis of metabolic syndrome but they do not reflect the human disease sufficiently. *Psammomys obesus*, a desert gerbil species, is able to subsist on a halophilic plants-based hypocaloric diet. However, in the presence of a relatively high energy regimen such as the standard laboratory diet, this sand rat develops T2DM [10, 11]. Indeed, the potential toxicity of glucose from this exogenous source (high but normal if compared to Wistar rats) will be drastically amplified, leading to a rapid evolution of the disease. Interestingly, prevention of this hyperglycemic state together with an enhancement of hepatic insulin sensitivity was found in diabetes-prone *Psammomys obesus* after exercise training [12]. Through this peculiar behavior, this kind of rodent represents an appropriate biological tool to uncover the features of nutritional diabetes. In addition, a previous experiment with perifused hepatocytes of *Psammomys* reported oxygen consumption rates smaller than those measured from Wistar rats. As well, levels of ATP and ADP were markedly lower in these gerbils [13].

In the light of these above considerations, the aim of the present work was to monitor in parallel the mitochondrial functioning and oxidative damage in diabetes-prone *Psammomys obesus*, by measuring the respiration intensity related to electron transfer chain complexes activities as well as some oxidative stress parameters after 18 weeks of treatment.

## 2. Materials and Methods

### 2.1. Animals and Diet

The Algerian sand rats *Psammomys obesus* used for this investigation were housed in suitable cages under controlled temperature and light conditions. Adult animals of both sexes (80–100 g) were divided into two groups: the control group consuming their plants-(*Salsola-foetida-*) based natural food, with a low energy diet (20 kcal/day) but rich in water and minerals—and the group fed the standard laboratory diet of high caloric value (32.5 kcal/day). Food and water were supplied during 18 weeks. Each animal was monitored for body weight and blood glucose in order to select *Psammomys *having a glycaemia superior to 100 mg/dL. All experimental procedures were authorized by the Institutional Animal Care Committee.

### 2.2. Biochemical Analysis

Fasted* Psammomys *rats were killed by cervical dislocation at the end of treatments, without anesthesia to avoid any further stress, and blood samples were collected in EDTA tubes. Plasma glucose and lipids (triglycerides, total cholesterol, HDL-cholesterol, LDL-c), hepatic and renal function markers (alanine transaminase (ALT), aspartate transaminase (AST), creatinine, urea) were measured by a spectrophotometric method adapted on a Cobas Mira automatic analyser. Plasma insulin was determined by radioimmunoassay. Extraction of hepatic total lipids was carried out using a process earlier described [14], and they were gravimetrically measured.

### 2.3. Oxidative Stress Assessment

Plasma total antioxidant status (TAS) was analysed in blood samples, using a commercial kit (Randox Laboratories LTD, UK). The principle of this assay is based on the reaction of peroxydase and H_2_O_2_ with the substrate azinodiethyl-benzothiazoline sulfonic acid (ABTS) to produce a radical cation of stable blue-green colour which was detected at 600 nm. The antioxidant capacity was inversely proportional to this coloration intensity and was expressed as mmol/L.

Lipid peroxidation was estimated from liver homogenates by measuring levels of malondialdehyde (MDA) through the thiobarbituric acid reactive substances method [15].

Liver GSH content was assayed using a commercially available kit (Cayman Chemical Company), based on the reaction with the thiol-specific reagent dithionitrobenzoic acid (DTNB). In this procedure, the sulfhydryl group of GSH reacts with DTNB to form a product which is reduced by glutathione reductase for recycling GSH and producing more thionitro-benzoic acid (TNB). The rate of TNB production was directly proportional to GSH levels, which were expressed as *μ*moles/mg protein.

The protein content of hepatic samples was determined according to the Lowry method [16], using bovine serum albumin (BSA) solution as a standard.

### 2.4. Mitochondria Isolation

Liver mitochondria from both *Psammomys* groups were prepared according to a standard differential centrifugation procedure, with all steps carried out at 4°C. After killing the animals, livers were quickly excised, rinsed, and chopped into an isolation medium (250 mM sucrose, 20 mM Tris-HCl, 1 mM EGTA, pH 7.4). The homogenates were centrifuged at 800 g for 10 min to remove nuclei and cell debris. Mitochondria were obtained from the supernatant by spinning twice at 8000 g for 10 min, and the pellet was resuspended in 0.5 mL of isolation buffer, then kept on ice. After measuring protein concentrations as above described, final mitochondrial suspensions were used immediately for respiration or stored at –80°C until enzyme analysis.

### 2.5. Oxidative Phosphorylation Measurement

Mitochondrial respiration was recorded polarographically, using a sealed oxygraphy chamber equipped with a Clark oxygen electrode and magnetic stirring. Oxygen consumption rates (*J*O_2_) were determined at 37°C in a respiration buffer (125 mM KCl, 20 mM Tris-HCl, 1 mM EGTA, 5 mM Pi) with FFA-BSA to avoid the presence of uncoupled mitochondria. The non-phosphorylating state 2 was initiated by the addition of either 5 mM glutamate/2.5 mM malate (G/M) or 5 mM succinate/0.5 mM malate (S/M) in presence of 1.25 *μ*M rotenone. The phosphorylating state 3 was obtained after addition of 1 mM ADP while state 4 was measured with 1.25 *μ*g/mL oligomycin, a specific inhibitor of ATP synthase. For uncoupled respiration, 75 *μ*M of dinitrophenol (DNP) was added, while cytochrome c oxidase activity was indirectly evaluated with 1 mM TMPD/5 mM ascorbate. The efficiency of oxidative phosphorylation was then assessed by the state 3-to-state 4 ratio, also called respiratory control index (RCR).

### 2.6. Electron Transfer Chain Activity and Cytochromes Content

Activities of respiratory complexes were assayed via slight changes of the protocols described by Malgat et al., using liver mitochondrial particles resulting from freezing-thawing cycles [17]. Complex I was assayed as the rate of NADH oxidation at 340 nm in 50 mM KPi buffer containing 3.75 mg/mL BSA, 100 *μ*M decylubiquinone, 100 *μ*M NADH. Rotenone (10 *μ*M) was specifically used to inhibit this complex whose real activity was deduced from difference between NADH oxidation without and with rotenone. Complex II was measured as the rate of 2,6-dichloro-indophenol (DCIP) reduction at 600 nm in 50 mM KPi buffer supplemented with 2.5 mg/mL BSA, 9.3 *μ*M antimycin A, 5 *μ*M rotenone, 100 *μ*M DCIP, 30 mM succinate. Enzyme activity was calculated between the difference before and after addition of 50 *μ*M decylubiquinone. Complex III was assayed by measuring the reduction of cytochrome c at 550 nm, with and without 9.1 *μ*M antimycin A. Isolated mitochondria were incubated in 100 mM KPi medium with 1 mg/mL BSA, 50 *μ*M EDTA, 1 mM KCN, 100 *μ*M oxidized cytochrome c, and the reaction was started by addition of 105.6 *μ*M decylubiquinol.

In parallel, the content in different cytochromes of the electron transfer chain was measured by dual-wavelength spectrophotometry, comparing the spectra of fully oxidized versus fully reduced cytochromes [18].

### 2.7. Statistical Analysis

All data were reported as mean ± SEM. Differences between both rat groups were determined by Student's *t*-tests, with a *P* value of either <0.05, <0.01, or <0.001 considered as statistically significant.

## 3. Results

### 3.1. Long-Term Metabolic Effects of High Caloric Diet and Impact on the Liver Redox State

**Figure 1:**
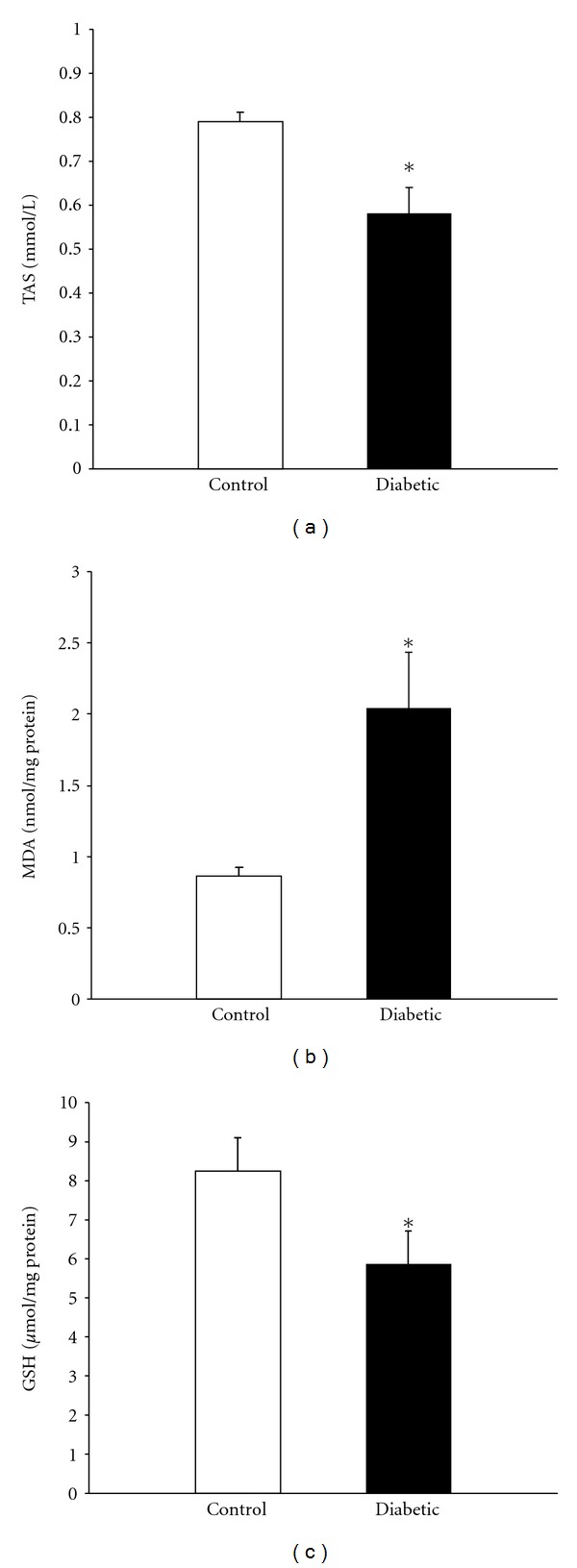
Oxidative stress in plasma and liver tissue. Total antioxidant defences or TAS (a), as well as the intrahepatic contents in MDA (b) and GSH (c), was, respectively, measured in fresh plasma and frozen liver homogenate of control or treated *Psammomys*. *P < 0.05 versus control group.

*Psammomys* rats fed a high caloric chow for 18 weeks developed a metabolic syndrome, with significant changes in their body weight (*P* < 0.05), glycemia (*P *< 0.01), and insulinemia (*P *< 0.001) as compared with those of control group (Table 1). Plasma lipids levels, in particular triglyceridemia and cholesterolemia, were also altered. Furthermore, diabetic *Psammomys* exhibited a severe liver deterioration, as evidenced by a substantial increase of transaminases activity (*P *< 0.05), the hepatic mass accretion together with tissue accumulation of triglyce-rides. A renal injury, characterized by a rise in uremia and creatininemia, was besides showed. On the other hand, such harmful conditions markedly decreased plasma antioxidant capacity (Figure 1), whereas index of lipid peroxidation simultaneously increased (Figure 1). In addition, a depletion in hepatic GSH was found (Figure 1), and the resulting oxidative stress status in the liver, as the GSH/GSSG ratio, was largely diminished by the hypercaloric diet (1.07 ± 0.82 versus 4.29 ± 0.69 for control animals, *P *< 0.01).

### 3.2. Oxygen Consumption

**Figure 2:**
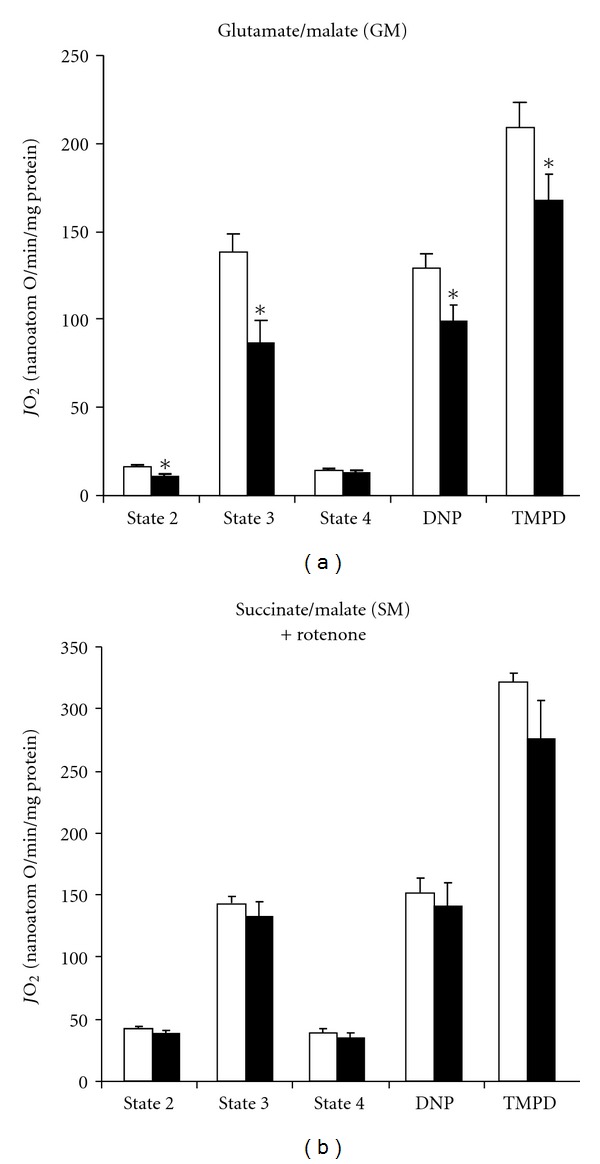
Mitochondrial respiration in *Psammomys*. Oxygen consumption rates (*J*O_2_) were assayed on mitochondria freshly isolated from control (open bars) or treated *Psammomys* (black bars), in the presence of glutamate/malate (a) or succinate/malate with rotenone (b) as energizing substrates. Various respiratory states were next assessed following the addition of different drugs. *P < 0.05 versus control group.

With mitochondria isolated from diabetic *Psammomys* livers, we noticed a net decline of the respiratory chain activity (Figure 2). Indeed, both basal state 2 and ADP-stimulated state 3 were significantly lower in mitochondria respiring on G/M (−24 and −31%, resp.) but barely decreased with S/M plus rotenone (−8 and −7.5%, resp.). The assessment of oligomycin-induced state 4 showed no modification whatever the substrates. These values led to a decreased RCR (state 3-to-state 4 ratio) for mitochondria only energized with G/M (Table 2). An inhibition of either DNP-uncoupled or TMPD/ascorbate-activated respirations (−25 and −19% resp.) was still observed under this particular condition, suggesting an alteration of some respiratory fluxes which could alter the oxidative phosphorylation machinery.

### 3.3. Mitochondrial Complexes Activity

**Figure 3:**
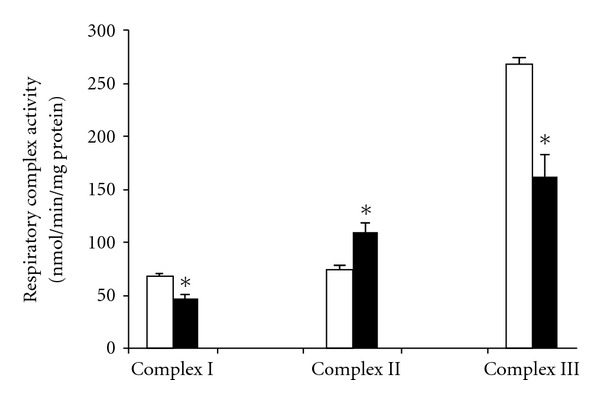
Activities of respiratory complexes I, II, III in liver mitochondria freshly isolated from control (open bars) or treated *Psammomys* (black bars). *P < 0.05 versus control group.

To assess whether the above respiration data were directly linked to some defects inside the electron transfer chain, enzyme activities of complexes I, II, III, combined with the level in different cytochromes, were measured in broken liver mitochondria. Complexes I and III were substantially decreased (−32 and −40% resp.) in organelles from diabetic animals as compared to control group, yet complex II unexpectedly increased by 42.4% (Figure 3). Interestingly, a smaller content in cytochrome aa3 was found in diabetic *Psammomys* liver mitochondria (Table 3), a result rather consistent with a reduced activity of cytochrome oxidase that we had above seen through the TMPD-dependent respiration.

## 4. Discussion

In this work, we have confirmed that *Psammomys obesus* is a reliable biological support for the study of metabolic disorders such as insulin resistance or T2DM, and whose the etiology is similar to its manifestation in humans. Our results are in accordance with studies using the Israeli *Psammomys* [19] or the Nile rat [20], even though the latter displayed less pronounced metabolic disturbances than *Psammomys obesus* after feeding a calorie-rich diet for 18 weeks. It may be underlined that the increment in hepatic mass-body weight ratio was positively correlated with hyperinsulinemia and tissue accumulation of lipids, meaning a profound liver injury similar to that reported by others [21]. A high proportion of soluble transaminases was also seen. Knowing that these enzymes are released when hepatocellular damage occurs [22] and, on the other side, that an incomplete oxidation due to chronic fuel excess can be linked to the inability for mitochondria to maintain sufficient ATP levels [23, 24], it seems that these deleterious metabolic defects are consistently associated with a drastic endogenous oxidative stress and subsequent mitochondrial dysfunction, mainly at the inner membrane activity level.

The current work studied oxidative phosphorylation capacity using liver mitochondria isolated from *Psammomys obesus*. Because there existed no or few relevant data about bioenergetics in these rats, it became quite difficult to compare our findings with those of recent literature. We, therefore, tried to discuss them at best with respect to other experimental models involving a mitochondrial dysfunction. Mitochondria from diabetes-prone *Psammomys obesus* showed a lower respiratory rate than that of control group: the decreases in both state 3 (with ADP) and uncoupled state indicate a loss of oxidative capacity. Similar results were obtained from Wistar rats fed a high-fat diet [25, 26], while no modifications or even higher respiration intensities were found with Zucker rats [27] or GK rats of 6 months age [3]. Cytochrome oxidase, last component of the respiratory chain, is well recognized as a controlling step of nonphosphorylating oxygen consumption [28]. Its activity, when indirectly determined in the presence of TMPD/ascorbate, was lower in diabetic *Psammomys obesus* compared to control animals, and this response was moreover accompanied by a significant loss in cytochrome aa3. Such an observation could reflect a smaller mitochondrial efficiency as evidenced by the reduction of RCR though this fact concerned more particularly the NAD-driven respiration (with glutamate/malate). These findings would be also partially explained by diet-induced changes in membrane lipids composition but this phenomenon merits to be further explored.

High glucose or free fatty acids flux or both impairs metabolic flexibility, which may enhance mitochondrial substrate supply and ROS production [7]. As the harmful effects of ROS on tissues are widely appreciated, the severity of diabetic state for *Psammomys obesus* is likely related to an exacerbated oxidative stress. An increase in lipoperoxidation together with a decline in TAS were reported. In line with these alterations, a decrease of GSH/GSSG ratio was clearly indicative of an impaired liver antioxidant system. Our data are in agreement with several studies using streptozotocin-treated rats [29, 30], rats fed a high fructose diet [31], or diabetic mice [32, 33]. In view of that an increased oxidative stress is also consistent with a slowing-down of oxidative phosphorylation, our bioenergetics results suggest the existence of multiple damaged sites along the electron transfer chain. Indeed, mitochondria from diabetic *Psammomys* revealed an inhibition of complex I and complex III, attributable to an excess in mitochondrial ROS as earlier suggested [34]. Another important finding of this work is that *Psammomys* fed a calorie-rich diet exhibited a net activation of complex II despite a nearly decrease of *J*O_2_ in presence of succinate (FAD-linked substrate), but with comparable RCR between diabetic and control rats. In support of our data, Cunningham et al. observed decreases in all respiratory complexes, except complex II, using liver biopsies from patients with steatosis [35]. Otherwise, complexes II and IV activities were augmented in GK rats or streptozotocin-induced diabetic rats [36].

A large number of studies invoked the involvement of ROS in the pathogenesis of mitochondrial DNA-related disorders [37, 38], and lipid peroxidation products can damage the mitochondrial genome [39]. One may infer that an increasing oxidative stress linked to the diabetic state should lead to mitochondrial DNA damages, altering the function of complexes I and III. Taken into account that the proteins of complex II encoded by nuclear genes are probably spared, any improvement of its activity can alleviate the abnormality or loss of other respiratory parameters, and determine the overall oxidative capacity. Interestingly enough, another kind of adaptative response of mitochondrial metabolism to a high glucose milieu was revealed in pancreatic islets from diabetic *Psammomys obesus* [40]. In this regard, the desert gerbil model could set up metabolic and/or molecular adjustments to circumvent some of the deleterious outcomes of insulin resistance.

It is concluded that a high-caloric diet causes metabolic troubles at the hepatic level in the *Psammomys obesus* rat after an 18-week treatment, and these changes are likely associated with a vast mitochondrial dysfunctioning. The present work is in agreement with an altering expression of oxidative phosphorylation genes, possibly resulting from aggravated oxidative stress, which justifies the performance of further studies to identify some molecular processes responsible for that mitochondrial impairment, and to quantify their relative influence for the liver.

## Figures and Tables

**Table 1:** Body weight and biochemical parameters of control (*n* = 10) and high caloric diet-fed *Psammomys* rats (*n* = 15). Each run was performed in duplicate. *P < 0.05, **P < 0.01, ***P < 0.001 versus control rat group (natural food).

Parameter	Control (18 weeks)	Diabetic (18 weeks)
Initial body wt (g)	87.8 ± 3.3	85 ± 3.9
Final body wt (g)	121.5 ± 4.1	138.5 ± 8.7*
Hepatic mass/body wt ratio (%)	3.4 ± 0.09	4.05 ± 0.12*
Glucose (mg/dL)	65.2 ± 0.03	267 ± 0.5**
Insulin (pmol/L)	166.5 ± 1.8	1439 ± 3.3***
Triglycerides (mg/dL)	74.6 ± 19	253 ± 45.4**
Cholesterol (mg/dL)	61 ± 4.2	145.7 ± 4.9**
HDL-C (mg/dL)	44.2 ± 3.3	48.5 ± 7.2
LDL-C (mg/dL)	17.8 ± 1.3	29.5 ± 3.2*
Hepatic total lipids (mg/100 g wet wt)	3720 ± 90	4900 ± 190*
Hepatic triglycerides (mg/100 g wet wt)	291 ± 19	654 ± 79**
Hepatic cholesterol (mg/100 g wet wt)	273 ± 11	322 ± 21*
Urea (mg/dL)	53.8 ± 4.6	73.6 ± 6.9*
Creatinine (mg/dL)	0.28 ± 0.05	0.45 ± 0.02*
ALT (U/L)	76 ± 8.4	136 ± 19**
AST (U/L)	86 ± 6.7	167 ± 25**

**Table 2:** Effect of dietary treatment on respiratory control ratios (RCR) of liver mitochondria from both *Psammomys* groups. *P < 0.05 versus control group.

		Oxygen consumption rate (natoms O/min/mg protein)				
	Glutamate/Malate (GM)			Succinate/Malate (SM) + rotenone		
	State 3	State 4	RCR	State 3	State 4	RCR
Control	138.8 ± 9.9	14.8 ± 0.6	9.4 ± 0.9	143.1 ± 5.3	39.3 ± 3.0	3.7 ± 0.3
Diabetic	96.1 ± 7.3*	14.5 ± 0.6	6.6 ± 0.2*	133.0 ± 12.3	35.4 ± 3.5	3.8 ± 0.2

**Table 3:** Effect of dietary treatment on levels in different cytochromes of liver mitochondria from both *Psammomys* groups.*P < 0.05 versus from control group.

	Cytochromes content (pmols/mg protein)			
	*a + a3*	*b*	*c1*	*c*
Control	66.1 ± 3.1	184.2 ± 6.0	95.0 ± 9.2	63.2 ± 21.0
Diabetic	51.3 ± 2.8*	219.4 ± 12.0	79.8 ± 7.9	58.2 ± 7.2
